# Postoperative 30-Day Comparative Complications of Multilevel Anterior Cervical Discectomy and Fusion and Laminoplasty for Cervical Spondylotic Myelopathy: An Evidence in Reaching Consensus

**DOI:** 10.3390/diagnostics13122024

**Published:** 2023-06-10

**Authors:** Ryan Wing-Yuk Chan, Yung-Hsiao Chiang, Hsiu-Chen Lin, Chih-Yau Chang, Yi-Syue Tsou

**Affiliations:** 1 Department of Neurosurgery, Taipei Medical University Hospital, Taipei 11031, Taiwan; b101095151@tmu.edu.tw (R.W.-Y.C.); ychiang@tmu.edu.tw (Y.-H.C.); 2 Taipei Neuroscience Institute, Taipei Medical University, Taipei 11031, Taiwan; 3 Department of Surgery, School of Medicine, Taipei Medical University, Taipei 11031, Taiwan; 4 Department of Pediatrics, School of Medicine, College of Medicine, Taipei Medical University, Taipei 11031, Taiwan; jane2@tmu.edu.tw; 5 Department of Clinical Pathology, Taipei Medical University Hospital, Taipei 11031, Taiwan; 6 Department of Quality Management, Taipei Medical University Hospital, Taipei 11031, Taiwan; 175241@h.tmu.edu.tw; 7 Ph.D. Program in Medical Neuroscience, College of Medical Science and Technology, Taipei Medical University, Taipei 11031, Taiwan

**Keywords:** multilevel anterior cervical discectomy and fusion (ACDF), laminoplasty (LAMP), cervical spondylotic myelopathy (CSM), propensity score matching (PSM)

## Abstract

Although a few large-scale studies have investigated multilevel anterior cervical discectomy and fusion (ACDF) and laminoplasty (LAMP) and their related complications for cervical spondylotic myelopathy (CSM), the optimal surgical intervention remains controversial. Therefore, we compared their 30 days of postoperative complications. Through the 2010–2019 ACS NSQIP Participant Use Data Files, we estimated the risk of serious morbidity, reoperation, readmission, mortality, and other postoperative complications. Initially, propensity score matching (PSM) of the preoperative characteristics of both groups was performed for further analysis. Multivariable logistic regression analysis provided OR and 95% CI for comparative complications. After PSM, 621 pairs of cohorts were generated for both groups. Increased frequency of postoperative complications was observed in the LAMP group, especially for surgical wound infection, no matter whether superficial (ACDF/LAMP = 0%/1.13%, *p* = 0.0154) or deep wound infection (ACDF/LAMP = 0%/0.97%, *p* = 0.0309). The mean length of total hospital stays (ACDF/LAMP = 2.25/3.11, *p* < 0.0001) and days from operation to discharge (ACDF/LAMP = 2.12/3.08, *p* < 0.0001) were longer, while the hospitalization rate for over 30 days (ACDF/LAMP = 4.67%/7.41%, *p* = 0.0429) and unplanned reoperation (ACDF/LAMP = 6.12%/9.34%, *p* = 0.0336) were higher in LAMP. Results also indicated congestive heart failure as a risk factor (adjusted OR = 123.402, *p* = 0.0002). Conclusively, multilevel ACDF may be a safer surgical approach than LAMP for CSM in terms of perioperative morbidities, including surgical wound infection, prolonged hospitalization, and unplanned reoperation. However, these approaches showed no significant differences in systemic complications and perioperative mortality.

## 1. Introduction

Cervical spondylotic myelopathy (CSM) is an age-dependent and degenerative disease of the cervical spine, which is characterized by the narrowing of the spinal canal and cord compression caused by facet joint hypertrophy, osteophytes, herniated discs, hypertrophic ligamentum flavum, and ossified posterior longitudinal ligament [1]. Surgical decompression, instead of conservative management, is indeed the only treatment choice during severe neurological deterioration [2]. In particular, multilevel anterior cervical decompression and fusion (ACDF) and laminoplasty (LAMP) represent two major treatments; however, the optimal surgical intervention remains controversial.

Multilevel ACDF provides anterior decompression and fusion for the cervical spine, while LAMP provides nonfusion and posterior decompression [3]. A few large-scale studies exist that have attempted to investigate complications arising from these two surgical approaches for the specific etiology of CSM. Besides, the results of the studies are inconsistent or even contradictory [4,5,6,7,8,9]. In a meta-analysis, ACDF demonstrated increased complications compared with LAMP; however, compared with the baseline, the Cobb angle of C2–C7 was reduced [10]. In another study, both ACDF through mini-incision and LAMP are successful treatments for long-level cervical spondylosis; nevertheless, it was found that ACDF through mini-incision is advantageous as it causes minimal trauma, resulting in lesser bleeding, and allows for faster recovery [11]. Moreover, it is also beneficial for reconstructing cervical lordosis.

Therefore, we conducted this observational study using the American College of Surgeons National Surgical Quality Improvement Program Participant Use Data Files (ACS NSQIP PUF) to compare the 30-day perioperative complications of multilevel ACDF versus LAMP for the treatment of CSM. Propensity score matching (PSM) analysis was used to minimize the selection bias in these surgical procedures. We characterized the incidence and predictors of individual complications, as well as the outcome measures of hospital length of stay (HLOS) and discharge destination by surgical cohort.

## 2. Materials and Methods

### 2.1. Database and Patient Selection

**Figure 1:**
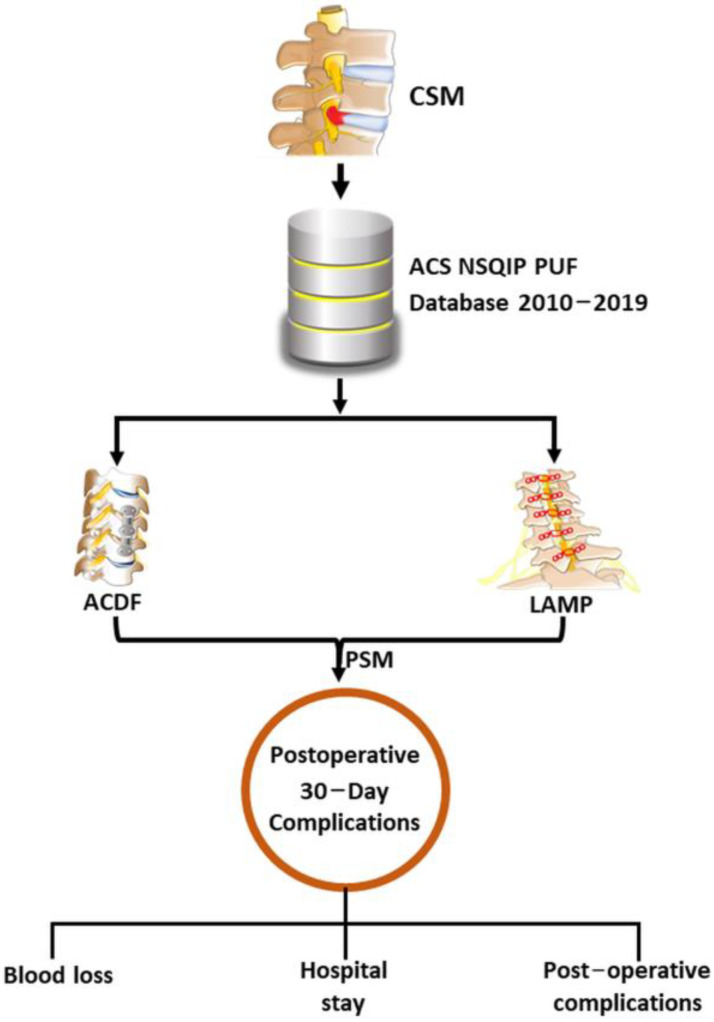
Schematic of study design. PSM: ACDF: anterior cervical discectomy and fusion, LAMP: laminoplasty, PSM: propensity score matching.

The overall schematic design of the study is represented in Figure 1. Briefly, all patients undergoing neurosurgical procedures in the American College of Surgeons (ACS) National Surgical Quality Improvement Program (NSQIP) database between 2010 and 2019 were included in the analysis. The ACS NSQIP prospectively collects data on over 200 variables about patient characteristics, comorbid conditions, operative details, and 30-day postoperative outcomes for different surgical procedures.

The data are collected by trained surgical/clinical reviewers using a thorough review process comprising chart/electronic medical review and careful follow-up to record outcomes. Due to a strict data collection process, the data are known to have an inter-reviewer disagreement rate of below 2% [12]. More information about ACS the NSQIP is available at http://www.acsnsqip.org accessed on 19 April 2023.

**Figure 2:**
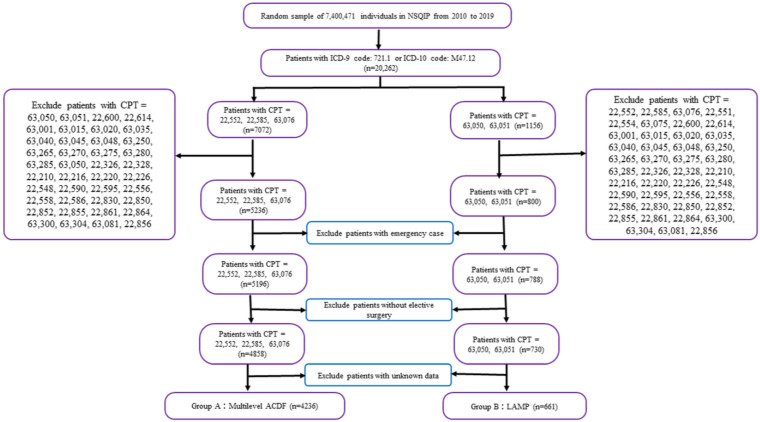
Flowchart of the study population. CPT: Current Procedural Terminology, ACDF: anterior cervical discectomy and fusion, LAMP: laminoplasty.

Records were filtered using the International Classification of Diseases 9th Edition (ICD-9) and 10th Edition (ICD-10) diagnosis codes to identify patients with the CSM registry between 2010 and 2019 (Figure 2). Current Procedural Terminology (CPT) codes were used to query the dataset for patients undergoing multilevel ACDF (22,552, 22,585, 63,076) and laminoplasty (63,050, 63,051). The average number of fused segments/levels in ACDF cases was ≥2, while the number of plastic laminae in LAMP cases was ≥2. Patients undergoing combined anterior and posterior spinal approaches and other surgical procedures were excluded from the cohort. Patients with missing data, emergency cases, and elective surgery were excluded from the study.

### 2.2. Statistical Analysis

PSM for the preoperative characteristic of the multilevel ACDF and LAMP groups was performed before further analysis. The paired *t*-test and McNemar test were used to evaluate significant differences between these groups. Multivariable logistic regression analysis provided odds ratios (OR) and 95% confidence intervals (CI) for complications in these two groups, which were adjusted for age, gender, BMI, diabetes mellitus with oral agents or insulin, open wound/wound infection, steroid use for a chronic condition, bleeding disorders, preoperative transfusion of ≥1 unit of whole/packed RBCs in 72 h before surgery, systemic sepsis, hypertension requiring medication, congestive heart failure (CHF) in 30 days before surgery, acute renal failure, being currently on dialysis, dyspnea, history of severe chronic obstructive pulmonary disease (COPD), ventilator dependence, disseminated cancer, being a current smoker within 1 year, wound classification, ASA classification, and selected laboratory values. All outcome variables were considered significant if they had a two-sided *p*-value < 0.05 from multivariate analyses. All statistical analysis was performed using the Statistical Analysis System (SAS) 9.1 package (SAS Institute Inc., Cary, NC, USA).

## 3. Results

In total, 4897 CSM patients were included in the analysis (Figure 1). PSM was conducted to balance the baseline covariates with the covariates of age, sex, comorbidities, region, and race in a 1:1 ratio using the exact matching technique to produce two equal cohorts of 621 patients. This cohort revealed that the LAMP group was found to be significantly male (ACDF/LAMP = 48.63%/66.34%, *p* < 0.0001) and older age (ACDF/LAMP = 60.69/61.86, *p* = 0.0496). Meanwhile, patients in the ACDF group showed a trend of significantly higher BMI (ACDF/LAMP = 30.72/29.57, *p* = 0.0027), preoperative dyspnea (ACDF/LAMP = 7.24%/2.74%, *p* = 0.0004), and lower preoperative hematocrit (ACDF/LAMP = 40.79/41.86, *p* < 0.0001) compared with the LAMP group (Table 1). 

We further assessed the perioperative complications and reoperation in the ACDF and LAMP groups (Table 2).

Increased perioperative complications were observed in the LAMP group, especially for surgical wound infection, no matter whether superficial (ACDF/LAMP = 0%/1.13%, *p* = 0.0154) or deep wound infection (ACDF/LAMP = 0%/0.97%, *p* = 0.0309). However, the occurrence of systemic infections included sepsis and septic shock with no significant difference between these groups. In the LAMP group, we found the mean length of total hospital stay (ACDF/LAMP = 2.25/3.11, *p* < 0.0001) and mean days from operation to discharge (ACDF/LAMP = 2.12/3.08, *p* < 0.0001) were longer than multilevel ACDF. Notably, the rate of hospitalization over 30 days (ACDF/LAMP = 4.67%/7.41%, *p* = 0.0429) and unplanned reoperation (ACDF/LAMP = 6.12%/9.34%, *p* = 0.0336) were also found higher for LAMP. However, the operation time was longer for multilevel ACDF than LAMP (ACDF/LAMP = 158.60/149.51, *p* = 0.0122). No significant difference was observed between the groups for the perioperative mortality rates. The preoperative patient condition and congestive heart failure but not operation type were closely related to the mortality rate (adj. OR = 123.402, *p* = 0.0002) (Table 3).

## 4. Discussion

In recent years, though fewer studies have contributed to decision making about the appropriate surgical management of CSM, the data remain controversial, and no consensus has yet been reached. Both ACDF and LAMP are well-established procedures for CSM. ACDF is an efficacious approach for direct neural decompression through rectifying cervical kyphotic alignment and preserving cervical spine stability [13]. Meanwhile, LAMP manages multilevel CSM through indirect decompression by the augmentation of the spinal canal [14]. In a study by Liu et al., ACDF required a shorter operation time than LAMP (187.78 vs. 115.92 min). On the contrary, our results revealed significantly shorter operative time in the LAMP group (158.60 vs. 149.51 min, *p* = 0.0122) [9]. Besides, some of the previous reports demonstrated no significant difference in operation time between these approaches [4,7,15]. Moreover, the number of operating segments was not specified. Contrary to the above reports on operation time, the previous studies documented higher blood loss in the LAMP group than in multilevel ACDF [7,9,15]. Although this result corresponds to our data on the higher blood loss in the LAMP group (ACDF/LAMP = 0.81%/1.61%, *p* = 0.194), it showed no significant difference.

Further, the reported ACDF complications include stenosis of the respiratory tract by swelling of the surrounding tissue, swallowing disturbance, and hoarseness due to traction of the vocal cord [16]. Meanwhile, the aftermath of laminoplasty surgery complications can lead to persistent neuropathic arm pain, axial neck pain, and a decline in cervical alignment, primarily caused by the advancement of kyphosis [17]. In terms of perioperative complications, most of the results of previous studies on LAMP and multilevel ACDF were reported as either inconsistent or contradictory. In some studies, the overall complications were significantly more frequent [4,5,8,9], while few of them revealed no significant difference in the complication rate as well between these surgical approaches [7,15]. In a study by Morishita et al., increased systemic complications, such as respiratory disease, cardiovascular events, and sepsis, were observed in the multilevel ACDF group, whereas local infection was frequent in the LAMP group [6]. This study is in agreement with our study, revealing frequent overall complications in the LAMP group. These complications include surgical wound problems, such as superficial wound infection and deep wound infection. Compared with multilevel ACDF, wound disruptions have been observed more frequently in the LAMP group, though the difference is not significant. This might be attributed to the use of the posterior approach, which is associated with generally larger and deeper wounds with an increased amount of soft tissue dissection. On the contrary, previous studies revealed an exceedingly low incidence of postoperative infection in ACDF with a mean rate of 0.07% compared with 6.0%–18.2% in posterior spine surgeries [18,19,20]. Even though surgical site infection is more frequent in the LAMP group, we did not observe any significant difference in the occurrence of systemic infections, including sepsis and septic shock. The higher rate of postoperative infection in the LAMP group might be attributable to the higher rate of unplanned reoperation, hospitalization over 30 days, and longer time of stay in the hospital in a short duration. However, in the long-term follow-up studies, instrument complications, such as pseudarthrosis and reconstruction failure, are much more common in the ACDF group (incidence rate = 36.5%)^3^ than in the LAMP group [4,5,8,9]. This may be ascribed to a higher reoperation rate in the ACDF group than the LAMP group in the long-term follow-up. Another study by Lee et al. found 36.3% and 9.1% incidence complications rate and revision rate, respectively, in the ACDF group [5].

Although the incidence of perioperative complications of LAMP for CSM patients is higher, this approach is not a risk factor for postoperative mortality. This result is consistent with a previous study by Morishita et al. [6]; however, our study revealed that congestive heart failure (CHF) may be a risk factor for postoperative mortality. Various previous studies have demonstrated heart failure as an established risk factor for postoperative mortality [21,22,23,24] not only for inpatient surgery but also for ambulatory noncardiac surgery [22]. The mortality risk progressively increases with the severity of heart failure [21,22]. In addition to perioperative mortality, CHF also increases complications, including reoperation, readmission, cardiac arrest, unplanned intubation, and ventilator requirement > 48 h [24]. Although the exact mechanism is not clear, these complications may be associated with an excessive amount of fluid administration and poor cardiac output during the perioperative period [25]. Therefore, the importance of perioperative care for heart failure patients should be underscored to reduce perioperative mortality and complications.

Apart from various positive outcomes, this study also includes some limitations. Though hospitals may follow patients longer, the ACS NSQIP PUF database retains the follow-up data of only 30-day postoperative mortality and morbidity outcomes; hence, preoperative disease severity and duration details may not be available. Moreover, the analysis includes only acute complications, whereas the data on long-term complications, such as the occurrence of adjacent segment disease for ACDF and alteration in kyphotic angle and intractable neck pain for the LAMP group, could not be excluded. For multilevel ACDF, the specific complications may include dysphagia, recurrent laryngeal nerve palsy, pseudarthrosis, Horner syndrome, vertebral artery injury, adjacent segment disease, esophageal perforation, and pseudoarthrosis [26]. Meanwhile, the complications associated with LAMP may include postoperative kyphosis and loss of motion, postoperative neck pain, and recurrent stenosis [27]. This database was extracted by diagnosis and surgical coding, which does not reflect radiological data, cervical alignment, and severity of heart failure; however, this information could influence the decision on the surgical method chosen. Besides, it is difficult to clearly distinguish CSM from other diseases, such as congenital stenosis and posterior longitudinal ligament (OPLL), through coding. The decision on surgical methods largely depends on the surgeon’s experience and preference. This bias could not be eliminated even after PSM. Besides, there are still differences existing in preoperative characteristics between the two groups even after PSM. Moreover, the database also lacked information about quality of life and cost-effectiveness, which is also important in choosing surgical methods. Despite these limitations, we have presented a large-scale study investigating perioperative complications of ACDF and LAMP for the treatment of CSM.

## 5. Conclusions

LAMP is associated with a higher incidence of surgical wound infection than multilevel ACDF in the short term after the operation. However, no significant difference between these approaches was observed in the rate of systemic complications or postoperative mortality.

## Figures and Tables

**Table 1:** Baseline patient characteristics between multilevel ACDF and LAMP before and after PSM. PSM: propensity score matching, ACDF: anterior cervical discectomy and fusion, LAMP: laminoplasty, BMI: bone mineral density, ASA: American Society of Anesthesiologists, COPD: chronic obstructive pulmonary disease. CHF: congestive heart failure. * *p* < 0.05.

	Before PSM			After 1:1 PSM		
Preoperative Characteristics	Multilevel ACDF (Group A)	LAMP (Group B)	*p*-Value	Multilevel ACDF (Group A)	Laminoplasty (Group B)	*p*-Value
*n*	4236	661	X	621	621	X
Mean age (std)	59.00 (10.80)	62.85 (10.93)	<0.0001 *	60.69 (10.65)	61.86 (10.37)	0.0496 *
Gender, male (%)	2141 (50.54)	431 (65.20)	<0.0001 *	302 (48.63)	412 (66.34)	<0.0001 *
Mean BMI (std)	30.51 (6.73)	29.46 (6.46)	0.0002 *	30.72 (6.98)	29.57 (6.46)	0.0027 *
Diabetes mellitus with oral agents or insulin (%)	0 = No: 3417 (80.67)	0 = No: 520 (78.67)	0.4830	0 = No: 489 (78.74)	0 = No: 493 (79.39)	0.9607
	1 = non-insulin: 537 (12.68)	1 = non-insulin: 93 (14.07)		1 = non-insulin: 87 (14.01)	1 = non-insulin: 84 (13.53)	
	2 = insulin: 282 (6.66)	2 = insulin: 48 (7.26)		2 = insulin: 45 (7.25)	2 = insulin: 44 (7.09)	
Open wound/wound infection (%)	13 (0.31)	5 (0.76)	0.0842	3 (0.48)	5 (0.81)	0.7257
Steroid use for chronic condition (%)	174 (4.11)	29 (4.39)	0.7373	20 (3.22)	21 (3.38)	0.8738
Bleeding disorders (%)	64 (1.51)	12 (1.82)	0.5557	10 (1.61)	9 (1.45)	0.8172
Preop transfusion of ≥1 unit of whole/packed RBCs in 72 h prior to surgery (%)	0 (0)	0 (0)	X	0 (0)	0 (0)	X
Systemic sepsis (%)	0 = none: 4226 (99.76)	0 = none: 660 (99.85)	1.0000	0 = none: 620 (99.84)	0 = none: 620 (99.84)	1.0000
	1 = SIRS: 10 (0.24)	1 = SIRS: 1 (0.15)		1 = SIRS: 1 (0.16)	1 = SIRS: 1 (0.16)	
	2 = sepsis: 0 (0)	2 = sepsis: 0 (0)		2 = sepsis: 0 (0)	2 = sepsis: 0 (0)	
	3 = septic shock: 0 (0)	3 = septic shock: 0 (0)		3 = septic shock: 0 (0)	3 = septic shock: 0 (0)	
Mean preoperative WBC (std)	7.40 (2.41)	7.15 (2.09)	0.0043 *	7.34 (2.70)	7.15 (2.12)	0.1708
Mean preoperative hematocrit (std)	41.56 (4.22)	41.48 (4.39)	0.6569	40.79 (3.94)	41.86 (4.06)	<0.0001
Hypertension requiring medication (%)	2368 (55.90)	408 (61.72)	0.0050 *	369 (59.42)	376 (60.55)	0.6852
CHF in 30 days before surgery (%)	14 (0.33)	4 (0.61)	0.2909	2 (0.32)	4 (0.64)	0.6867
Mean preoperative serum creatinine (std)	0.94 (0.53)	0.99 (0.58)	0.0433 *	0.92 (0.33)	0.97 (0.52)	0.0656
Acute renal failure (preop) (%)	1 (0.02)	0 (0)	1.0000	0 (0)	0 (0)	X
Currently on dialysis (preop) (%)	16 (0.38)	4 (0.61)	0.3349	1 (0.16)	1 (0.16)	1.0000
Dyspnea (%)	0 = no: 3949 (93.22)	0 = no: 643 (97.28)	0.0003 *	0 = no: 576 (92.75)	0 = no: 604 (97.26)	0.0004 *
	1 = moderate exertion: 272 (6.42)	1 = moderate exertion: 18 (2.72)		1 = moderate exertion: 43 (6.92)	1 = moderate exertion: 17 (2.74)	
	2 = at rest: 15 (0.35)	2 = at rest: 0 (0)		2 = at rest: 2 (0.32)	2 = at rest: 0 (0)	
History of severe COPD (%)	243 (5.74)	33 (4.99)	0.4404	43 (6.92)	30 (4.83)	0.1168
Ventilator dependence (%)	0 (0)	0 (0)	X	0 (0)	0 (0)	X
Disseminated cancer (%)	6 (0.14)	3 (0.45)	0.1101	0 (0)	0 (0)	X
Current smoker within 1 year (%)	1104 (26.06)	147 (22.24)	0.0361 *	136 (21.90)	145 (23.35)	0.5416
Wound classification (%)	1 = clean: 4222 (99.67)	1 = clean: 656 (99.24)	0.2079	1 = clean: 620 (99.84)	1 = clean: 616 (99.19)	0.2176
	2 = clean/contaminated: 13 (0.31)	2 = clean/contaminated: 5 (0.76)		2 = clean/contaminated: 1 (0.16)	2 = clean/contaminated: 5 (0.81)	
	3 = contaminated: 0 (0)	3 = contaminated: 0 (0)		3 = contaminated: 0 (0)	3 = contaminated: 0 (0)	
	4 = dirty/infected: 1 (0.02)	4 = dirty/infected: 0 (0)		4 = dirty/infected: 0 (0)	4 = dirty/infected: 0 (0)	
ASA classification (%)	1 = no disturb: 86 (2.03)	1 = no disturb: 7 (1.06)	0.0852	1 = no disturb: 5 (0.81)	1 = no disturb: 7 (1.13)	0.3877
	2 = mild disturb: 1890 (44.62)	2 = mild disturb: 272 (41.15)		2 = mild disturb: 254 (40.90)	2 = mild disturb: 267 (43.00)	
	3 = severe disturb: 2150 (50.76)	3 = severe disturb: 365 (55.22)		3 = severe disturb: 340 (54.75)	3 = severe disturb: 234 (53.78)	
	4 = life threat: 110 (2.60)	4 = life threat: 17 (2.57)		4 = life threat: 22 (3.54)	4 = life threat: 13 (2.09)	
	5 = moribund: 0 (0)	5 = moribund: 0 (0)		5 = moribund: 0 (0)	5 = moribund: 0 (0)	

**Table 2:** Postoperative complications before and after PSM. PSM: propensity score matching. SSI: surgical site infection, CPR: cardiopulmonary resuscitation. * *p* < 0.05.

	Before PSM					After 1:1 PSM				
Postoperation Occurrence	Multilevel ACDF (Group A)	LAMP (Group B)	*p*-Value	B versus AOR (95% CI)	*p*-Value of OR	Multilevel ACDF (Group A)	LAMP (Group B)	*p*-Value	B versus AOR (95% CI)	*p*-Value of OR
*n*	4236	661	X	X	X	621	621	X	X	X
Mean total operation time (std)	159.32 (68.93)	149.96 (58.02)	0.0002 *	0.835 (0.724, 0.962)	0.0128 *	158.60 (69.40)	149.51 (57.64)	0.0122 *	0.837 (0.690, 1.015)	0.0712
Superficial surgical site infection (%)	5 (0.12)	7 (1.06)	0.0003 *	9.057 (2.866, 28.621)	0.0002 *	0 (0)	7 (1.13)	0.0154 *	>999.999 (<0.001, >999.999)	0.9531
Deep incisional SSI (%)	3 (0.07)	6 (0.91)	0.0003 *	12.925 (3.225, 51.807)	0.0003 *	0 (0)	6 (0.97)	0.0309 *	>999.999 (<0.001, >999.999)	0.9341
Organ space SSI (%)	5 (0.12)	0 (0)	1.0000	<0.001 (<0.001, >999.999)	0.9702	0 (0)	0 (0)	X	X	X
Wound disruption (%)	2 (0.05)	5 (0.76)	0.0007 *	16.136 (3.124, 83.339)	0.0009 *	0 (0)	5 (0.81)	0.0620	>999.999 (<0.001, >999.999)	0.9398
Pneumonia (%)	38 (0.90)	7 (1.06)	0.6606	1.183 (0.526, 2.659)	0.6851	8 (1.29)	7 (1.13)	0.7950	0.874 (0.315, 2.424)	0.7952
Unplanned intubation (%)	34 (0.80)	1 (0.15)	0.0783	0.187 (0.026, 1.370)	0.0990	7 (1.13)	1 (0.16)	0.0694	0.141 (0.017, 1.153)	0.0677
Ventilator > 48 h (%)	26 (0.61)	1 (0.15)	0.1652	0.245 (0.033, 1.811)	0.1683	5 (0.81)	1 (0.16)	0.2176	0.199 (0.023, 1.706)	0.1407
Progressive renal insufficiency (%)	1 (0.02)	0 (0)	1.0000	<0.001 (<0.001, >999.999)	0.9702	0 (0)	0 (0)	X	X	X
Acute renal failure (%)	4 (0.09)	1 (0.15)	0.5158	1.603 (0.179, 14.364)	0.6732	0 (0)	0 (0)	X	X	X
Urinary tract infection (%)	24 (0.57)	11 (1.66)	0.0048 *	2.970 (1.448, 6.098)	0.0030 *	5 (0.81)	10 (1.61)	0.1940	2.016 (0.685, 5.934)	0.2028
Pulmonary embolism (%)	18 (0.42)	6 (0.91)	0.1247	2.147 (0.849, 5.429)	0.1063	2 (0.32)	6 (0.97)	0.2875	3.019 (0.607, 15.015)	0.1770
Cardiac arrest requiring CPR (%)	4 (0.09)	2 (0.30)	0.1887	3.211 (0.587, 17.565)	0.1785	0 (0)	2 (0.32)	0.4996	>999.999 (<0.001, >999.999)	0.9426
Myocardial infarction (%)	9 (0.21)	1 (0.15)	1.0000	0.712 (0.090, 5.626)	0.7476	4 (0.64)	1 (0.16)	0.3740	0.249 (0.028, 2.232)	0.2140
Return to OR (%)	60 (1.42)	14 (2.12)	0.1691	1.506 (0.837, 2.710)	0.1720	9 (1.45)	13 (2.09)	0.3895	1.454 (0.617, 3.426)	0.3922
DVT/thrombophlebitis (%)	11 (0.26)	4 (0.61)	0.1333	2.338 (0.742, 7.366)	0.1467	2 (0.32)	3 (0.48)	1.0000	1.502 (0.250, 9.020)	0.6563
Bleeding transfusions (%)	15 (0.35)	14 (2.12)	<0.0001 *	6.089 (2.926, 12.674)	<0.0001*	5 (0.81)	10 (1.61)	0.1940	2.016 (0.685, 5.934)	0.2028
Sepsis (%)	16 (0.38)	2 (0.30)	1.0000	0.801 (0.184, 3.489)	0.7671	1 (0.16)	2 (0.32)	1.0000	2.003 (0.181, 22.149)	0.5709
Septic shock (%)	7 (0.17)	2 (0.30)	0.3480	1.834 (0.380, 8.845)	0.4502	1 (0.16)	1 (0.16)	1.0000	1.000 (0.062, 16.023)	1.0000
Mean length of total hospital stay (std)	2.03 (2.83)	3.22 (2.40)	<0.0001 *	5.005 (4.300, 5.825)	<0.0001 *	2.25 (3.86)	3.11 (2.23)	<0.0001 *	5.215 (4.190, 6.491)	<0.0001 *
Mean days from operation to discharge (std)	1.96 (2.39)	3.18 (2.35)	<0.0001 *	5.127 (4.403, 5.969)	<0.0001 *	2.12 (2.94)	3.08 (2.22)	<0.0001 *	5.328 (4.278, 6.635)	<0.0001 *
Still in hospital > 30 days (%)	217 (5.12)	49 (7.41)	0.0157 *	1.483 (1.075, 2.045)	0.0163 *	29 (4.67)	46 (7.41)	0.0429 *	1.633 (1.012, 2.636)	0.0446 *
Unplanned reoperation (%)	275 (6.49)	62 (9.38)	0.0064 *	1.491 (1.117, 1.990)	0.0067 *	38 (6.12)	58 (9.34)	0.0336 *	1.581 (1.033, 2.418)	0.0348 *
Death (%)	13 (0.31)	3 (0.45)	0.4672	1.481 (0.421, 5.211)	0.5406	0 (0)	3 (0.48)	0.2494	>999.999 (<0.001, >999.999)	0.9533

**Table 3:** Postoperative mortality before and after PSM. PSM: propensity score matching. CHF: congestive heart failure.

**Before PSM**				
Parameter	Adjusted OR	95% CI		*p*-Value
Age	1.11	1.049	1.174	0.0003 *
Preoperative hematocrit	0.873	0.803	0.949	0.0014 *
Preoperative serum creatinine	0.36	0.12	1.083	0.0690
Steroid use for chronic condition	4.74	1.315	17.086	0.0174 *
CHF in 30 days before surgery	15.267	0.92	253.327	0.0572
Currently on dialysis (preop)	191.335	12.017	>999.999	0.0002 *
Dyspnea: moderate exertion vs. no	0.362	0.021	6.325	0.4863
Dyspnea: at rest vs. no	26.253	2.45	281.345	0.0069 *
Disseminated cancer	16.523	2.333	117.02	0.0050 *
**After PSM**				
CHF in 30 days before surgery	123.402	9.577	>999.999	0.0002 *

* *p* < 0.05.

## Data Availability

Data are publicly available at ACS NSQIP at http://www.acsnsqip.org accessed on 19 April 2023.

## References

[1] Young W.F. (2000). Cervical spondylotic myelopathy: A common cause of spinal cord dysfunction in older persons. Am. Fam. Physician.

[2] Hartig D., Batke J., Dea N., Kelly A., Fisher C., Street J. (2015). Adverse events in surgically treated cervical spondylopathic myelopathy: A prospective validated observational study. Spine.

[3] Hirai T., Yoshii T., Arai Y., Sakai K., Torigoe I., Maehara H., Tomori M., Taniyama T., Sato H., Okawa A. (2017). A Comparative Study of Anterior Decompression with Fusion and Posterior Decompression with Laminoplasty for the Treatment of Cervical Spondylotic Myelopathy Patients with Large Anterior Compression of the Spinal Cord. Clin. Spine Surg..

[4] Yoshii T., Egawa S., Chikuda H., Wakao N., Furuya T., Kanchiku T., Nagoshi N., Fujiwara Y., Yoshida M., Taguchi T. (2021). A systematic review and meta-analysis comparing anterior decompression with fusion and posterior laminoplasty for cervical spondylotic myelopathy. J. Orthop. Sci..

[5] Lee J.J., Lee N., Oh S.H., Shin D.A., Yi S., Kim K.N., Yoon D.H., Shin H.C., Ha Y. (2020). Clinical and radiological outcomes of multilevel cervical laminoplasty versus three-level anterior cervical discectomy and fusion in patients with cervical spondylotic myelopathy. Quant. Imaging Med. Surg..

[6] Morishita S., Yoshii T., Okawa A., Fushimi K., Fujiwara T. (2020). Comparison of Perioperative Complications Between Anterior Decompression with Fusion and Laminoplasty For Cervical Spondylotic Myelopathy: Propensity Score-matching Analysis Using Japanese Diagnosis Procedure Combination Database. Clin. Spine Surg..

[7] Chen Q., Qin M., Chen F., Ni B., Guo Q., Han Z. (2019). Comparison of Outcomes Between Anterior Cervical Decompression and Fusion and Posterior Laminoplasty in the Treatment of 4-Level Cervical Spondylotic Myelopathy. World Neurosurg..

[8] Hirai T., Yoshii T., Sakai K., Inose H., Yamada T., Kato T., Kawabata S., Arai Y., Shinomiya K., Okawa A. (2018). Long-term results of a prospective study of anterior decompression with fusion and posterior decompression with laminoplasty for treatment of cervical spondylotic myelopathy. J. Orthop. Sci..

[9] Liu T., Yang H.L., Xu Y.Z., Qi R.F., Guan H.Q. (2011). ACDF with the PCB cage-plate system versus laminoplasty for multilevel cervical spondylotic myelopathy. J. Spinal Disord. Tech..

[10] Xu L., Sun H., Li Z., Liu X., Xu G. (2017). Anterior cervical discectomy and fusion versus posterior laminoplasty for multilevel cervical myelopathy: A meta-analysis. Int. J. Surg..

[11] Zhang Y., Yang G., Zhou T., Chen Y., Gao Z., Zhou W., Gu Y. (2022). Efficacy and safety of anterior cervical discectomy and fusion (ACDF) through mini-incision and posterior laminoplasty (LAMP) for treatment of long-level cervical spondylosis: A retrospective cohort study. BMC Surg..

[12] Davenport D.L., Holsapple C.W., Conigliaro J. (2009). Assessing surgical quality using administrative and clinical data sets: A direct comparison of the University HealthSystem Consortium Clinical Database and the National Surgical Quality Improvement Program data set. Am. J. Med. Qual..

[13] Wang B., Lü G., Kuang L. (2018). Anterior cervical discectomy and fusion with stand-alone anchored cages versus posterior laminectomy and fusion for four-level cervical spondylotic myelopathy: A retrospective study with 2-year follow-up. BMC Musculoskelet. Disord..

[14] Mitsunaga L.K., Klineberg E.O., Gupta M.C. (2012). Laminoplasty techniques for the treatment of multilevel cervical stenosis. Adv. Orthop..

[15] Montano N., Ricciardi L., Olivi A. (2019). Comparison of Anterior Cervical Decompression and Fusion versus Laminoplasty in the Treatment of Multilevel Cervical Spondylotic Myelopathy: A Meta-Analysis of Clinical and Radiological Outcomes. World Neurosurg..

[16] Ghazwan H., Oscar L.A., Monjur A. (2022). Dysphagia Following Anterior Cervical Spine Surgery. Dysphagia.

[17] Riester M., Taylor J.M., Feifer A., Koppie T., Rosenberg J.E., Downey R.J., Bochner B.H., Michor F. (2012). Combination of a novel gene expression signature with a clinical nomogram improves the prediction of survival in high-risk bladder cancer. Clin. Cancer Res..

[18] Xu R., Bydon M., Sciubba D.M., Witham T.F., Wolinsky J.P., Gokaslan Z.L., Bydon A. (2011). Safety and efficacy of rhBMP2 in posterior cervical spinal fusion for subaxial degenerative spine disease: Analysis of outcomes in 204 patients. Surg. Neurol. Int..

[19] Ghobrial G.M., Harrop J.S., Sasso R.C., Tannoury C.A., Tannoury T., Smith Z.A., Hsu W.K., Arnold P.M., Fehlings M.G., Mroz T.E. (2017). Anterior Cervical Infection: Presentation and Incidence of an Uncommon Postoperative Complication. Glob. Spine J..

[20] Sebastian A., Huddleston P., Kakar S., Habermann E., Wagie A., Nassr A. (2016). Risk factors for surgical site infection after posterior cervical spine surgery: An analysis of 5,441 patients from the ACS NSQIP 2005–2012. Spine J..

[21] Cha Y.H., Ha Y.C., Ryu H.J., Lee Y.K., Park S.H., Lee K.J., Koo K.H. (2020). Effect of heart failure on postoperative short and long-term mortality in elderly patients with hip fracture. Injury.

[22] Lerman B.J., Popat R.A., Assimes T.L., Heidenreich P.A., Wren S.M. (2019). Association between Heart Failure and Postoperative Mortality Among Patients Undergoing Ambulatory Noncardiac Surgery. JAMA Surg..

[23] Hammill B.G., Curtis L.H., Bennett-Guerrero E., O’Connor C.M., Jollis J.G., Schulman K.A., Hernandez A.F. (2008). Impact of heart failure on patients undergoing major noncardiac surgery. Anesthesiology.

[24] Turrentine F.E., Sohn M.W., Jones R.S. (2016). Congestive Heart Failure and Noncardiac Operations: Risk of Serious Morbidity, Readmission, Reoperation, and Mortality. J. Am. Coll. Surg..

[25] Pradeep A., Rajagopalam S., Kolli H.K., Patel N., Venuto R., Lohr J., Nader N.D. (2010). High volumes of intravenous fluid during cardiac surgery are associated with increased mortality. HSR Proc. Intensive Care Cardiovasc. Anesth..

[26] Yee T.J., Swong K., Park P. (2020). Complications of anterior cervical spine surgery: A systematic review of the literature. J. Spine Surg..

[27] Weinberg D.S., Rhee J.M. (2020). Cervical laminoplasty: Indication, technique, complications. J. Spine Surg..

